# Rational formulation engineering of fraxinellone utilizing 6-O-α-D-maltosyl-β-cyclodextrin for enhanced oral bioavailability and hepatic fibrosis therapy

**DOI:** 10.1080/10717544.2021.1976310

**Published:** 2021-09-14

**Authors:** Jianbo Li, Tiange Feng, Weijing Yang, Yaru Xu, Shuaishuai Wang, Huijie Cai, Zhilei Liu, Hong Qiang, Jinjie Zhang

**Affiliations:** a Institute of Medical and Pharmaceutical Sciences, Zhengzhou University, Zhengzhou, Henan, China; b Henan Key Laboratory of Targeting Therapy and Diagnosis for Critical Diseases, School of Pharmaceutical Sciences, Zhengzhou University, Zhengzhou, Henan, China; c Collaborative Innovation Center of New Drug Research and Safety Evaluation, Zhengzhou, Henan, China

**Keywords:** Fraxinellone, cyclodextrin derivatives, oral bioavailability, solubility, hepatic fibrosis

## Abstract

Although Fraxinellone (Frax) isolated from *Dictamnus albus* L. possessed excellent anti-hepatic fibrosis activity, oral administration of Frax suffered from the inefficient therapeutic outcome *in vivo* due to negligible oral absorption. At present, the oral formulation of Frax is rarely exploited. For rational formulation design, we evaluated preabsorption risks of Frax and found that Frax was rather stable while poorly dissolved in the gastrointestinal tract (78.88 μg/mL), which predominantly limited its oral absorption. Further solubility test revealed the outstanding capacity of cyclodextrin derivatives (CDs) to solubilize Frax (6.8–12.8 mg/mL). This led us to study the inclusion complexes of Frax with a series of CDs and holistically explore their drug delivery performance. Characterization techniques involving ^1^H-NMR, FT-IR, DSC, PXRD, and molecular docking confirmed the most stable binding interactions when Frax complexed with 6-O-α-D-maltosyl-β-cyclodextrin (G_2_-β-CD-Frax). Notably, G_2_-β-CD-Frax exhibited the highest solubilizing capacity, fast dissolution rate, and superior Caco-2 cell internalization with no obvious toxicity. Pharmacokinetic studies demonstrated markedly higher oral bioavailability of G_2_-β-CD-Frax (5.8-fold that of free drug) than other Frax-CDs. Further, long-term administration of G_2_-β-CD-Frax (5 mg/kg) efficiently inhibited CCl_4_-induced hepatic fibrosis in the mouse without inducing any toxicity. Our results will inspire the continued advancement of optimal oral Frax formulations for anti-fibrotic therapy.

## 1. Introduction

Fraxinellone (Frax), an active ingredient isolated from *Cortex Dictamni*, has attracted enormous interest for its potent anti-fibrosis activity (Wu et al., 2016; Zheng et al., 2021). Recent studies showed that Frax can suppress excessive activation of hepatic stellate cells in the CCl_4_-induced hepatic fibrotic mice model by markedly modulating the secretion of pro-fibrotic factors and inhibiting CUG-binding protein 1 (CUGBP1) expression (Wu et al., 2016). More recent studies demonstrated that Frax can regulate the synthesis of extracellular matrix by targeting tumor-associated fibroblasts, thus strengthening the anti-tumor immune response (Xing et al., 2018; Pei et al., 2019; Chen et al., 2020). Unfortunately, low bioavailability strongly limits its utilization as an orally administered drug for fibrosis therapy, because of insufficient drug levels in plasma and undesired treatment outcomes (Ruan et al., 2006). Therefore, the development of a new oral Frax delivery platform with improved bioavailability is highly desired for the efficient treatment of fibrosis.

Elucidating the pre-absorption risks is a key prerequisite for the rational design of oral drug formulations (Ünal et al., 2020). Particularly, the physicochemical properties of drugs and their stability in the gastrointestinal tract are fundamental to the selection of oral formulation strategy (Sun et al., 2012; Jambhekar & Breen, 2013). For example, Tye et al. (2016) systemically evaluated the physicochemical properties of an eight-drug metabolic cocktail and thereby recommended the progression of its suitable oral formulations into a clinical validation study. Another example can be found in our previous study on the pre-absorption risks of Morin, one of the most important flavonoid compounds (Li et al., 2019). Morin showed poor oral bioavailability mainly owing to its high intestinal metabolism, suggesting that inhibiting the metabolism of Morin was critical to achieve maximal oral absorption. However, the fundamental physicochemical parameters of Frax within the gastrointestinal (GI) tract are still unclear, making its oral formulation poorly conceived and designed (Ran et al., 2007).

In the present study, our group assessed the pivotal pre-absorption risks of Frax and found that poor water solubility was a major absorption barrier to oral delivery of Frax. Solubility tests were therefore conducted using various solubilizers and demonstrated the superior solubilization capacity of cyclodextrin derivatives (CDs). Based on these findings, we reasoned that we might improve the oral bioavailability of poor-soluble Frax by complexing it with CDs. CDs are cone-shaped oligosaccharides typically composed of 6–8 glucopyranose units (Jambhekar & Breen, 2016; Jansook et al., 2018). The inclusion of drugs inside the cavity of CDs is considered a simple but attractive approach to address poor water solubility without impairing pharmacological activity (Lodagekar et al., 2019; Wang et al., 2020). Yan et al. have proved that β-cyclodextrin/Frax inclusion complex significantly improved the aqueous solubility while maintained the binding behavior of Frax to human serum albumin, a key transporter in the human body (Yan et al., 2016). To obtain optimal water solubility enhancement, hydrophilic CD derivatives, such as hydroxypropyl-β-cyclodextrin (HP-β-CD) and sulfobutyl ether β-cyclodextrin (SBE-β-CD), are often used (Nair et al., 2014; Devasari et al., 2015; Ren et al., 2019; Gratieri et al., 2020; Shankar et al., 2021). For example, SBE-β-CD has been utilized to help solubilize the lipophilic drug erlotinib and enhance its therapeutic effects (Devasari et al., 2015). More recently, the complex of HP-β-CD with the poorly soluble drug Naringenin achieved improved dissolution and potent anti-inflammatory effects at only 20% of the dose needed for the drug on its own (Gratieri et al., 2020). Another previous study reported that 20% HP-β-CD solution significantly improved oral bioavailability of Frax and concomitant liver injury protection, but complexation of HP-β-CD with Frax has not been explored (Ran et al., 2007). Recently, 6-O-α-D-maltosyl-β-cyclodextrin (G_2_-β-CD), as a newly developed CD, has shown excellent water solubility and low toxicity (Lucas-Abellán et al., 2008; Pinho et al., 2014; Li et al., 2015). However, specific information regarding the ability of G_2_-β-CD to improve oral drug absorption is still lacking.

Herein, we seek to enhance the oral bioavailability of Frax *via* inclusion complexation with a series of CD derivatives, β-CD, HP-β-CD, SBE-β-CD, and G_2_-β-CD. Stoichiometry, the apparent stability constant (*K_c_*), and the solubility improvement of CDs-Frax were determined by phase solubility studies. Related molecular mechanisms of CD-Frax complexation were systematically investigated using various techniques. Subsequently, the likely conformations of the complexes were explored by the construction of molecular models. The CDs-Frax were further investigated by *in vitro* drug release, Caco-2 cell internalization, and oral bioavailability studies. Finally, G_2_-β-CD-Frax was selected for therapeutic efficacy evaluation in CCl_4_-induced hepatic fibrotic rats.

## 2. Materials and methods

### 2.1. Materials

β-cyclodextrin (1134 g/mol, β-CD) and 2-hydroxypropyl-β-cyclodextrin (1319.6 g/mol, HP-β-CD) were obtained from Jiangsu Fengyuan Biochemical Technology (Jiangsu, China) and Zhiyuan Biotechnology (Shandong, China), respectively. Sulfobutyl ether-β-cyclodextrin (SBE-β-CD) with an average degree of sulfobutyl substitution of 6.6 (mean molecular weight was 2176.8 g/mol) was obtained from Zhiyuan Biotechnology (Shandong, China), while 6-O-alpha-D-maltosyl-β-cyclodextrin (G_2_-β-CD), a mono-maltose substituted derivative with an exact molecular weight of 1458.47 g/mol, was purchased from Shanghai Aladdin Biochemical Technology (Shanghai, China) (Mohtar et al., 2017; Yasmin et al., 2019). Fraxinellone (Frax, purity > 98%) was obtained from Pufei De Biotech (Chengdu, China). Cell culture supplies and Hydroxyproline assay kit were obtained from Solarbio Science & Technology (Beijing, China) and Nanjing Jiancheng Institute of Biological Engineering (Nanjing, China), respectively.

### 2.2. Preabsorption evaluation of Frax

#### 2.2.1. Aqueous solubility and log P measurement

The water solubility of Frax was determined as previously reported (Li et al., 2019). Briefly, excess amount of Frax was ultrasonically dispersed in water and placed in a shaker (120 rpm, 37 °C) for 3 days followed by centrifugation. Twenty microliters of supernatant were taken out from the samples and then diluted with methanol and filtration to detect drug concentration by a well-established HPLC method (see supporting information).

For octanol/water partition coefficient (Log *P*) measurements, a certain amount of Frax was dissolved in the PBS-saturated octanol (at pH 1.2, 4.5, and 6.8) followed by the addition of the same volume of octanol-saturated PBS. The mixtures were placed in an air bath shaking at 100 rpm at 37 °C for 24 h. The samples were then centrifuged (12,000 rpm, 10 min) for oil/water phase separation. The drug concentration in these two phases was analyzed by HPLC, respectively.

#### 2.2.2. Stability of Frax in artificial gastrointestinal (GI) fluid and homogenates

The stability studies were performed according to the previous study (Zhang et al., 2015). Briefly, 20 μL of DMSO solution of Frax (10 mg/mL) was added to 10 mL of simulated gastric fluid (SGF) or simulated intestinal fluid (SIF) containing pepsin/without pepsin. Afterward, the samples were placed in a shaker (100 rpm, 37 °C). At predetermined timepoints, a suitable amount of samples were withdrawn and mixed with methanol. After a thorough vortex, the mixture was centrifuged and the resulted supernatant was subjected to HPLC to determine Frax content.

GI segments (the stomach, duodenum, jejunum, ileum, and colon) were immediately isolated after the rats were sacrificed. Thereafter, the mucosa of each segment was homogenized and centrifuged following gently scraping. The supernatant was separated and subjected to BCA protein quantitative kit to adjust the final protein concentration to 1 mg/mL. DMSO solution of Frax (5 mg/mL) was added in the homogenate to achieve a final drug concentration of 20 μg/mL. After incubation at 37 °C for predetermined durations, samples were immediately precipitated by methanol, centrifuged, and injected into HPLC to measure the Frax concentration. The drug concentration measured before the incubation was set to 100%.

### 2.3. Solubility measurements of Frax in various solubilizers

Excess Frax was added into Solutol HS 15, Tween 80, PEG 400, Transcutol P, β-CD, SBE-β-CD, HP-β-CD, and G_2_-β-CD solution (50%), respectively. The samples were vortexed followed by incubation in a shaker (120 rpm, 37 °C) for 72 h. After the process, the samples were centrifuged to separate the supernatant containing dissolved Frax. After suitable dilution and filtration, Frax concentration in the supernatant was analyzed by the HPLC method.

### 2.4. Preparation of CD-Frax inclusion complexes

CDs-Frax complexes were prepared by a combination of ultrasonication and lyophilization techniques (Cui et al., 2019). Specifically, β-CD (0.31 g), G_2_-β-CD (0.4 g), HP-β-CD (0.36 g), or SBE-β-CD (0.6 g) were added to 1 mL of de-ionized water containing 10 mg Frax in separate tubes, respectively. The suspensions were thoroughly mixed and sonicated at 200 W in an ultrasonic bath for 0.5 h. After incubation in a shaker (100 rpm, 37 °C) for 72 h, undissolved residues were removed by centrifugation (4024.8 *g*, 10 min). The supernatants were freeze-dried (FreeZone Plus 2.5 L, Labconco, KS, USA) and stored in airtight containers at room temperature.

### 2.5. Characterizations of inclusion complexes

Phase solubility studies were conducted following a previously reported method (Cui et al., 2019). Frax was excessively mixed with increasing molar concentrations (0–350.00 mM) of each CD aqueous solution. The resulting mixtures were sonicated for 15 min, followed by shaking on a laboratory shaker (100 rpm, 37 °C) for 72 h. The samples were subjected to HPLC analysis after centrifugation (4024.8 *g*, 10 min). Phase solubility profiles were plotted by monitoring Frax concentration as a function of the molar concentration of each CD. Based on the phase solubility curves, we calculated the apparent stability constants (*K_c_*, L/mol) according to Equation (1):

(1) $$K_{c}=\frac{\text{Slope}}{S_{0}(1-\text{Slope})}$$

where *S*_0_ is the water solubility of Frax at equilibrium.

^1^H NMR experiments were performed on a 400 MHz NMR spectrometer (Ascend 400, Bruker, USA). Free Frax and CDs-Frax inclusion complexes dissolved in dimethyl sulfoxide (DMSO)-d_6_ were analyzed by ^1^H NMR. In addition, free Frax and CDs-Frax inclusion complexes were assessed in the solid-state using FTIR, P-XRD, SDT, and FSEM. All studies were performed following the procedures detailed in the Supplementary File.

### 2.6. Computational evaluation

To dock Frax into β-CD, SBE-β-CD, HP-β-CD, or G_2_-β-CD CDs for prediction of binding affinity and energetic properties, the commonly used computational method, molecular docking calculations was conducted using the software Molecular Operating Environment (CCGI, Montreal, Canada) (Matencio et al., 2021). The two-dimensional structures of Frax and CDs were converted into three-dimensional structures using an energy minimization algorithm in the software. Then the protonation state of the CDs and the direction of the hydrogens were optimized using LigX (pH 7, 300 K). Docking was performed using the MMFF94x: EHT force field and Reaction Field implicit solvation. The docking workflow followed the ‘induced fit’ protocol, in which the moieties of the binding site within the CD structure were allowed to adjust to the Frax conformation in a constrained manner, with a weighting factor of 10 to restrain CD atoms near their original positions. The various docked positions of Frax molecules were ranked based on London dG scoring. Finally, a force field refinement was applied to the top 30 positions, which were re-scored using GBVI/WSA dG.

### 2.7. *In vitro* drug release from CDs-Frax inclusion complexes

*In vitro* drug release performance was studied by a dialysis method in phosphate buffer (pH 6.8 and 7.4) and 0.1 M HCl (pH 1.2) at 37 °C. Briefly, a certain volume of Frax suspension, SBE-β-CD-Frax, G_2_-β-CD-Frax, and HP-β-CD-Frax dispersion solution were enclosed in 1500 Da dialysis bags, respectively. The dialysis bag was then transferred and immersed into the release medium under gentle shaking. After 0.5, 1, 1.5, 2, 2.5, 3.5, 4, and 6 h, 1 mL of the release media was aspirated and supplemented with 1 mL of fresh media. The Frax concentration in the samples was analyzed by HPLC. The cumulative drug release (%) was calculated with the following Equation (2) (Chuang et al., 2018):

(2) $$\text{Drug}\;\text{release}\;(\%)=\frac{V_{e}\sum_{1}^{n-1}C_{i}+V_{0}C_{n}}{m_{\text{Frax}}}$$

where *V*_0_ (mL) is the release media volume, *C_n_* (μg/mL) is the drug concentration in the sample, *V_e_* (mL) is the replaced medium volume, and *m*_Frax_ (mg) is the amount of drug in the sample.

### 2.8. Absorption evaluation of Frax in Caco-2 cells

Cytotoxicity effects and cellular uptake ability of Frax, CDs, and CDs-Frax complexes were examined using Caco-2 cells. For cytotoxicity evaluation, Caco-2 cells were incubated with the medium containing Frax, G2-β-CD-Frax, HP-β-CD-Frax, and SBE-β-CD-Frax at various drug concentrations (25–300 μg/mL) followed by cell viability evaluation by MTT assay (Li et al., 2019). Each CD solution was also used to determine whether the cell toxicity was caused by Frax or CDs. For cellular uptake studies, Caco-2 cells were treated with Frax and CDs-Frax (100 μg/mL) for 0.5, 1, and 2 h, respectively. For P-gp inhibition evaluation, the cells were pretreated with verapamil at a concentration of 100 μg/mL and further treated with each drug or formulation. Thereafter, the cells were washed and replenished with fresh PBS (300 μL). The cell lysate was prepared by three cycles of freeze-thaw. Protein concentration in the samples was quantified by the BCA kit while the Frax concentration was determined by HPLC.

### 2.9. Oral bioavailability study

Pharmacokinetic studies were conducted in Male Wistar rats (180–220 g) supplied by the Center of laboratory animals, Zhengzhou University. The Use Committee of Zhengzhou University (IRB0000861) approved the experimental protocol. In addition, all animal experiments were conducted according to the Guidelines of Animal Experimentation of the Laboratory Animal Center. Rats were acclimatized before experiments and provided free access to food and water.

Rats divided into five groups at random (*n* = 5) were dosed with the following formulations: intravenous administration of Frax (10 mg/mL) or orally given Frax suspension (150 mg/kg) or SBE-β-CD-Frax, HP-β-CD-Frax, and G_2_-β-CD-Frax (150 mg/kg), respectively. Blood (0.4 mL) was obtained by orbital sinus collection at 0.17, 0.25, 0.75, 1, 1.5, 2, 4, 6, 8, and 10 h post-administration. To determine Frax concentration in plasma, 200 μL methanol, 100 μL acetonitrile, and 20 μL 5% hydrochloric acid were added to rat plasma (100 μL) gradually. Following vortexing and centrifugation (4024.8 *g*, 10 min), the supernatant was collected, dried by air blowing at room temperature, and reconstituted with 150 μL of the mobile phase. The samples were sonicated and vortexed until homogeneity. After centrifugation, the supernatant (50 μL) was aspirated and injected into HPLC. The standard curve encompassed a linear range of 0.05–10 μg/mL and was used to calculate Frax concentration in plasma. Pharmacokinetic parameters were calculated using DAS software version 3.0 and reported as mean ± standard deviation. The oral absolute and relative bioavailability (*F_a_* and *F_r_*) were calculated using the following Equations (3) and (4):

(3) $$F_{a}(\%)=\frac{{\mathrm{AUC}}^{\mathrm{test}}{\mathrm{Dose}}^{i.v}}{{\mathrm{AUC}}^{i.v}{\mathrm{Dose}}^{\mathrm{test}}}$$

(4) $$F_{r}(\%)=\frac{{\text{AUC}}_{\text{test}}}{{\text{AUC}}_{\text{reference}}}$$

where AUC is the area under the pharmacokinetic curves

### 2.10. CCl_4_-induced liver fibrosis in mice, drug treatment, biochemical and histopathological examination

The Kunming male mice (weight of 28–30 g) were divided into 10 groups (*n* = 10): normal group, CCl_4_-induced liver fibrosis group, CCl_4_ induction treated with Frax or G_2_-β-CD-Frax treatment groups (5, 10, and 20 mg/kg, respectively), CCl_4_ induction with colchicine (0.2 mg/kg) treatment group, CCl_4_ induction with G_2_-β-CD solution treatment (equivalent to 20 mg/kg of G_2_-β-CD-Frax) group. The mice in the normal group were treated with olive oil only, while the other groups were intraperitoneally injected with 10% CCl_4_-olive oil every other week for 10 weeks (5 mL/kg for the first dose and 3 mL/kg for the second starting dose). Drug treatment began on the fourth week of modeling and continued every day for 7 weeks. One day after the last administration, blood in all the groups was immediately harvested for assays of AST and ALT. Rats were then sacrificed and livers were collected and divided into two parts for histological examination and Hydroxyproline (HYP) assay, respectively.

### 2.11. Safety evaluation of G_2_-β-CD-Frax for seven weeks

The Kunming male mice (weight of 28–30 g) were divided into 10 groups (*n* = 10): normal group, Frax or G_2_-β-CD-Frax treatment groups with a Frax dose of 5, 10, and 20 mg/kg, respectively, G_2_-β-CD solution treatment (equivalent to 20 mg/kg of G_2_-β-CD-Frax) group. Drug treatment continued every day for 7 weeks. One day after the last administration, blood and major tissues (heart, liver, spleen, lung, and kidney) were obtained from the blank group and drug-treated groups (20 mg/kg) for histological examination. The whole blood was directly analyzed using an automatic blood cell analyzer to obtain hematological parameters. The collected tissues were subjected to histological examination.

### 2.12. Statistical analysis

Intergroup differences were analyzed for significance by one-way ANOVA, and results were depicted by Graph Pad Prism 6.0 (GraphPad Software, USA). Significant differences were indicated by *p* < .05.

## 3. Results and discussion

### 3.1. Pre-absorption evaluation of Frax

**Figure 1.**
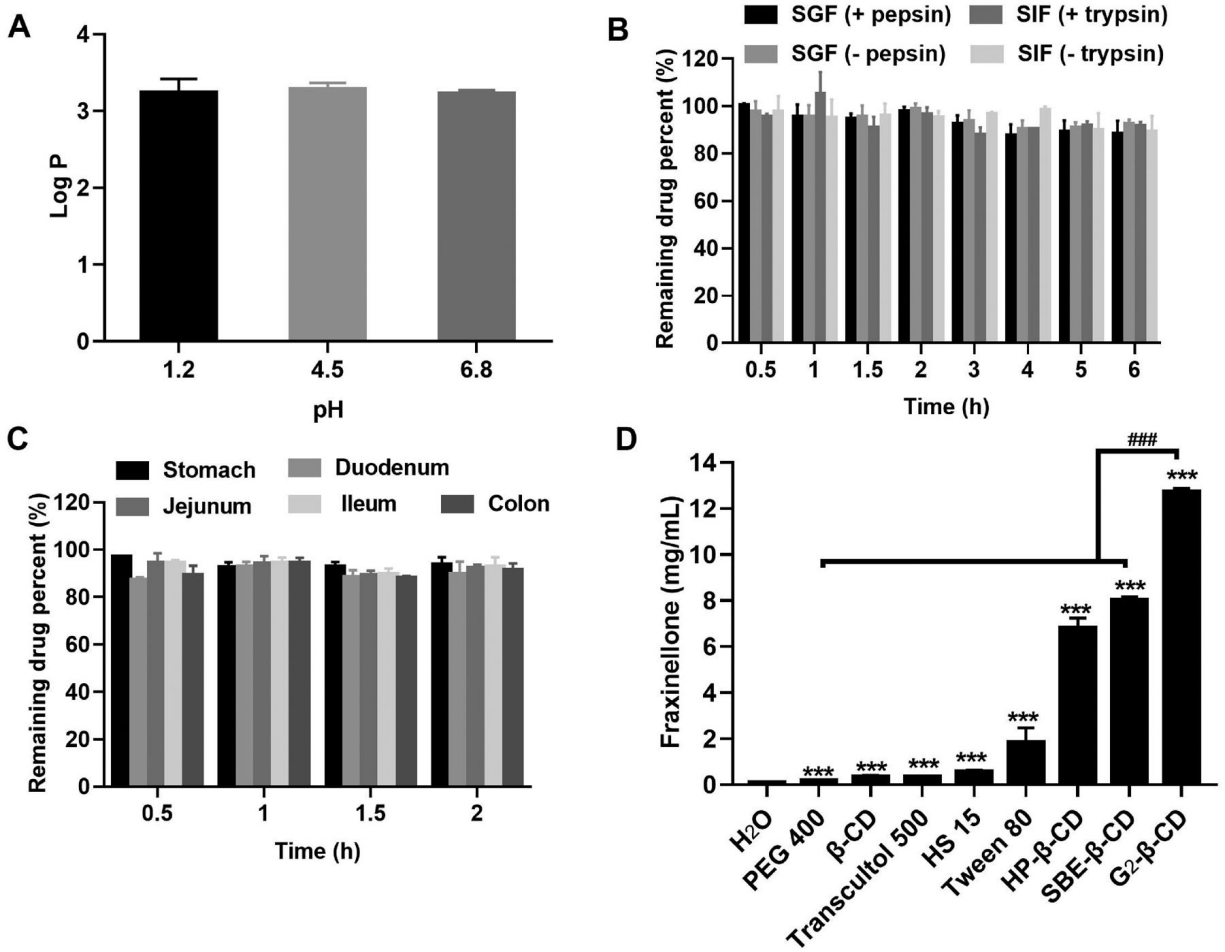
Preabsorption risk assessment of Fraxinellone (Frax). (A) Log *P* of Frax under different gastrointestinal pH conditions. (B) Stability of Frax after incubation with stimulated gastrointestinal fluids in the presence or absence of enzymes at 37 °C. (C) Stability of Frax in the homogenates of various gastrointestinal segments at 37 °C. (D) Solubility of Frax in 50% (w/v) solution of different solubilizers in water at 37 °C. ***p < .001, compared with the drug solubility in water. *^###^p* < .001, compared with G_2_-β-CD. Data are mean ± SD (*n* = 3 per group).

The orally administered drug will be present in a wide pH range along the GI tract, which potentially impacts their solubility (Koziolek et al., 2016). In the present study, we measured the aqueous solubility of Frax at 37 °C regardless of pH because Frax is completely non-ionized. The water solubility of Frax was extremely low (78.88 μg/mL). The partition coefficient between octanol and water (Log *P*) identifies the liposolubility of compounds, which is the key property in drug interaction with the human body (Bergström et al., 2016). Log *P* of Frax (3.2 ± 0.17) measured at different pH values showed no significant difference (Figure 1(A)), partially confirmed the pH-independent water solubility of Frax. Small molecular compounds having Log *P* > 3 are typically suggested to be solvation-limited and poorly absorbed (Tsopelas et al., 2017). In addition, drugs with a high Log *P* are also known to increase metabolic vulnerability (Ruan et al., 2006). Unexpectedly, Frax exerts negligible degradation in simulated GI fluids as well as GI homogenates as shown in Figures 1(B,C), suggesting good enzymatic stability in the GI tract. Combined with the previous finding that Frax has good intestinal permeability (Ruan et al., 2006), solubility enhancing formulation strategy is highly desired for sufficient absorption of Frax.

Further solubility tests were therefore performed to select good solubilizing excipients for Frax. The results showed that all excipients investigated herein exhibited a significant solubilizing effect on Frax (Figure 1(D)). Moreover, cyclodextrin derivatives (CDs) demonstrated a superior improvement in Frax solubility than the other solubilizers. Notably, Frax solubility in G_2_-β-CD solution (12.74 mg/mL) was highest among all the tested excipients, which was 160 times higher than that in water (0.079 μg/mL). Given this, we focused on the inclusion complexes of Frax with a series of CDs and systemically investigate their oral drug delivery performance in the subsequent studies to select efficient formulation for hepatic fibrosis therapy.

### 3.2. Characterization of CDs-Frax complex

#### 3.2.1. Stoichiometry (n) and stability constant (K_c_)

**Figure 2.**
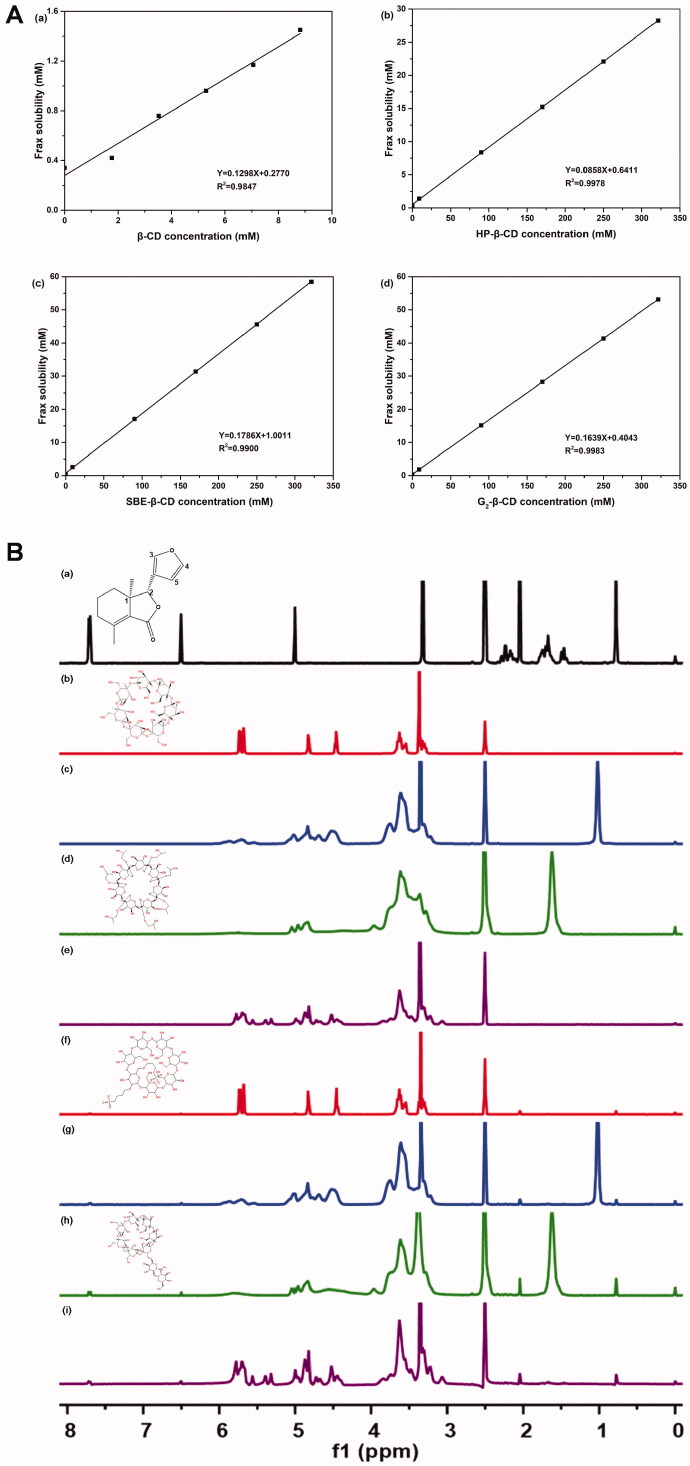
Characterization of CD-Frax complexes by phase solubility study and ^1^HNMR analysis. (A) Phase solubility diagrams of fraxinellone (Frax) at 37 °C in the presence of β-cyclodextrin (CD) (a), HP-β-CD (b), SBE-β-CD (c), or G_2_-β-CD (d). (B) Proton nuclear magnetic resonance (^1^H NMR) spectra of Frax (a), β-CD (b), HP-β-CD (c), SBE-β-CD (d), G_2_-β-CD (e), and the inclusion complexes β-CD-Frax (f), HP-β-CD-Frax (g), SBE-β-CD-Frax (h), and G_2_-β-CD-Frax (i).

Before performing a functional test of CDs-Frax complex *in vivo*, we first validated the formation of CDs-Frax complex and explored the underlying mechanism *via* various techniques. Phase solubility studies help us obtain the stoichiometric ratio and apparent stability constant (*K_c_*) in the complex (Aytac et al., 2016). The phase solubility behaviors of Frax in these CDs are shown in Figure 2(A). The stoichiometry was close to 1:1 in the assayed concentration range, based on the regression analysis of the phase solubility data. The linear phase solubility curve was therefore classified as A_L_-type (Aytac et al., 2016). Consistent with the findings in the solubility test, all these CDs were shown to improve the aqueous solubility of Frax at varying degrees. When complexed with 8.82 mM CDs, Frax solubility increased 3.93-fold in the case of β-CD (0.31 mg/mL), 4.18-fold in the case of HP-β-CD (0.33 mg/mL), 7.61-fold in the case of SBE-β-CD (0.60 mg/mL), and 5.45-fold in the case of G_2_-β-CD (0.43 mg/mL). Our result is in line with previous work reporting that β-CD showed limited water solubility at concentrations up to 8.82 mM and therefore did not solubilize drugs as well as other hydrophilic CD derivatives (Chi et al., 2015). Strikingly, when CD concentrations were 350 mM, Frax solubility increased 83.04-fold (6.55 mg/mL) with HP-β-CD, 171.91-fold (13.56 mg/mL) with SBE-β-CD and 156.19-fold (12.32 mg/mL) with G_2_-β-CD. These results suggest superior solubility improvement by SBE-β-CD and G_2_-β-CD. Previous work has also reported that SBE-β-CD can solubilize drugs (Jambhekar & Breen, 2016; Yildiz et al., 2017), and the present work goes further by demonstrating the great promise of G_2_-β-CD for the same purpose.

One thing that should be noted was that the mass percentage concentration of 350 mM SBE-β-CD and G_2_-β-CD aqueous solutions was calculated to be 76.1 and 51.5%, respectively. Generally, the drug loading capacity of cyclodextrin complexes is calculated by the mass ratio of drugs in the complex to the whole complex (Kfoury et al., 2017; Lima et al., 2019). In addition, most of the cyclodextrins derivatives have a wide molar mass distribution due to different degrees and positions of substituents (dos Santos Silva Araújo et al., 2021). Therefore, the solubilization capacity of CDs was evaluated by comparing the Frax solubility in the CDs of equal mass percentage concentration (see section 3.1), rather than molar concentration.

The excellent solubilization capacity of CDs inspired us to compare the *K_c_* values of these complexes. The *K_c_* value for Frax complexation at 37 °C was 109.34 M^−1^ for β-CD, 807.32 M^−1^ for HP-β-CD, 168.20 M^−1^ for SBE-β-CD, and 997.68 M^−1^ for G_2_-β-CD. That the *K_c_* values were in the range of 50–2000 M^−1^ suggests optimum interaction between the drug and CDs, which is highly relevant for improving oral bioavailability and therapeutic efficacy (Loftsson et al., 2005). However, it should be noted that G_2_-β-CD shows a markedly higher K_c_ value than SBE-β-CD, indicating its potential to form a stable inclusion complex with Frax in an aqueous solution (Tang et al., 2015).

#### 3.2.2. ^1^H NMR

NMR is an extremely powerful technique for validating the inclusion complex formation and analyzing host–guest chemistry in solution (Yang et al., 2010). Figure 2(B) shows the ^1^H NMR spectra of Frax, CDs, and CDs-Frax complex in DMSO-d_6_. Representative chemical shifts of protons in Frax before and after the formation of the complex are presented in Table S1. These upfield shifts indicate that Frax was embedded within the CD cavity when forming the complex (Rescifina et al., 2019). The chemical shifts were small probably because the Frax and CD interact non-covalently (Jambhekar & Breen, 2016). Indeed, this suggestion received support from the following characterization studies.

#### 3.2.3. FTIR

**Figure 3.**
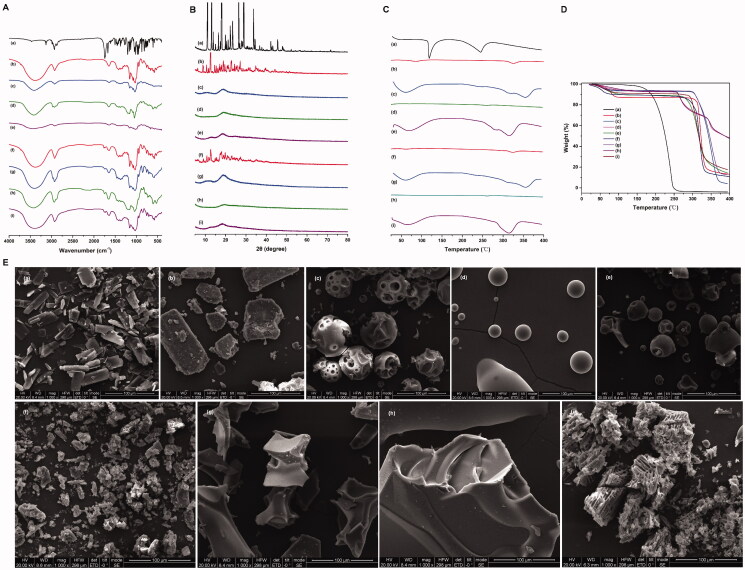
Characterization of CD-Frax complexes in the solid-state. Fourier-transform infrared spectra (FTIR) (A), powder X-ray diffraction pattern (P-XRD) (B), differential scanning calorimetry analysis (DSC) (C), thermal gravimetric analysis (TGA) (D) and field emission scanning electron microscopy analysis (FSEM) (E) of fraxinellone (Frax) (a), β-cyclodextrin (CD) (b), HP-β-CD (c), SBE-β-CD (d), G_2_-β-CD (e), and the inclusion complexes β-CD-Frax (f), HP-β-CD-Frax (g), SBE-β-CD-Frax (h), and G_2_-β-CD-Frax (i).

As shown from the FTIR spectra of free Frax (Figure 3(A,a)), Frax exhibited a significant characteristic peak at 1672.05 cm^−1^, assigned to C=C stretching vibrations in the furan ring. In addition, absorption of the C=O group in the lactone ring of Frax was observed at 1743.03 cm^−1^. In contrast, the inclusion complexes showed unique features in the FTIR spectra. For example, the stretching of the C=C peak at 1672.05 cm^−1^ in the furan ring of Frax shifted to 1646.2 cm^−1^ in β-CD-Frax, 1646.78 cm^−1^ in HP-β-CD-Frax, 1653.58 cm^−1^ in SBE-β-CD-Frax, and 1642.18 cm^−1^ in G_2_-β-CD-Frax, with this stretching peak much less intense in the complexes. These results demonstrate that the furan ring of Frax may be buried inside the CD cavity. In addition, the stretching of the C=O peak at 1743.03 cm^−1^ in the lactone ring of Frax shifted to 1762.702 cm^−1^ in β-CD-Frax, 1745.745 cm^−1^ in HP-β-CD-Frax, 1752.20 cm^−1^ in SBE-β-CD-Frax, and 1735.44 cm^−1^ in G_2_-β-CD-Frax. This shift suggests that the C=O group of Frax strongly interacts with the hydrogen-bond donor of CDs (Yang et al., 2010).

The FTIR spectra of all the CDs showed strong bond intensities at 3300–3500 cm^−1^, which was ascribed to the O–H stretching vibrations (Figure 3(A,b–e)). Complexation caused major changes in the –OH stretching peak of CDs (3300–3500 cm^−1^), indicating that –OH stretching vibration was disturbed by an interaction with Frax. In fact, nearly all the absorption peaks of CDs broadened and deepened in intensity as a result of complexation. This was likely because hydrogen bonding between Frax and CDs led to a larger dipole moment when the corresponding group vibrated. Overall, FTIR suggested that Frax was trapped within the cavities of CDs upon inclusion complex formation, probably through the interaction of the lactone and furan rings of Frax with the –OH of CDs.

#### 3.2.4. P-XRD

Frax exhibited several sharp characteristic peaks at diffraction angles (2*θ*) of 11.084, 13.123, 17.914, 18.199, 23.484, 26.466, and 28.953°, suggesting a crystalline nature (Figure 3(B,a)). β-CD exhibited peaks at diffraction angles (2θ) of 12.713, 18.916, 20.815, 21.281, 22.935, 25.628, and 27.177°, also indicating crystallinity (Figure 3(B,b)). The other CDs showed only one or two broad peaks, indicating an amorphous, non-crystalline state (Figure 3(B,c–e)) (Li et al., 2015). In contrast, the inclusion complexes did not show any of the diffraction peaks corresponding to Frax (Figure 3(B,f–i)). The diffraction patterns of CD-Frax shared the characteristic peaks of amorphous CDs, indicated that Frax lost its crystallinity when it formed a complex. Since amorphous solids are generally more soluble than crystalline ones (Bergström et al., 2016), we can conclude that the structural transformation of Frax after complexation is indispensable for its solubility enhancement.

#### 3.2.5. Thermal analysis

The DSC spectrum of Frax showed a characteristic melting endothermic peak at 116 °C, suggested that Frax existed in crystal forms (Figure 3(C,a)). Strikingly, this endothermic peak was completely absent from the thermograms of the CD-Frax complexes. DSC results corroborate the P-XRD findings showing the loss of crystallinity of Frax upon complex formation. TGA showed that CD-Frax lost mass at three stages of heating. In the first stage (75–100 °C), 10% mass loss from CD-Frax complexes occurred at about 90 °C and was similar to the mass loss from the corresponding free CDs, probably due to the loss of water from the CD cavity. In the second stage (270–310 °C), CD-Frax complexes lost about 80% (w/w) of their mass, probably because of Frax decomposition. Frax decomposition began around 160 °C for the free drug but at 265 °C when the drug was complexed with CDs (Figure 3(D,a)). The third mass loss from CD-Frax complexes was associated with the loss of water, and it occurred at a higher temperature than the corresponding loss of water from free CDs. These observations verify the inclusion of Frax within the cavity of CD complexes, which alters the drug’s dehydration stages and reduces its thermal stability.

#### 3.2.6. Morphological analysis

As observed by FSEM (Figure 3(E)), the morphology and size of the particles of pure Frax and CDs were completely different. Pure Frax (Figure 3(E,a)) appeared as rod-like particles, while β-CD exhibited in a larger form (Figure 3(E,b)). Consistent with previous studies, pure HP-β-CD, G_2_-β-CD, and SBE-β-CD were spherical with cavity-containing structures (Figure 3(E,c–e)) (Mohandoss et al., 2019). The morphology of Frax changed when it formed CDs-Frax complexes. β-CD-Frax appeared as smaller blocks under TEM, somewhat similar to the crystals of free Frax or β-CD (Figure 3(E,f)). Regarding all the other CDs-Frax complexes, we observed a new solid phase and loss of crystallinity, in consistent with our previous findings by P-XRD and thermal analysis (Figure 3(E,g–i)). Irregular bulky particles were observed, which further validated the inclusion of the drug into the CD structure.

### 3.3. Modeling of interactions between CDs and Frax in inclusion complexes

**Figure 4.**
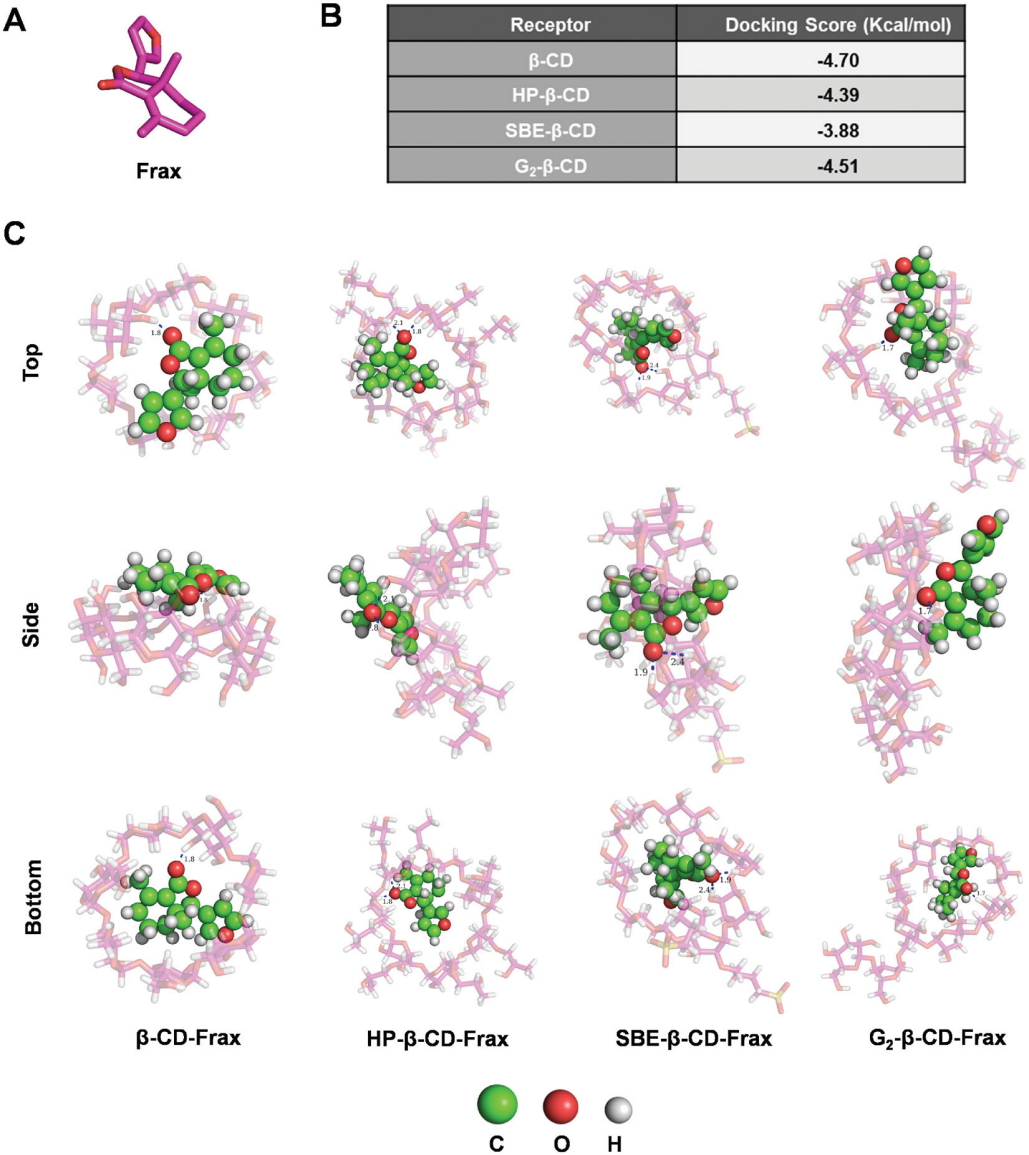
Docking studies to predict interactions between fraxinellone (Frax) and cyclodextrins (CDs) in inclusion complexes. (A) Structure of Frax. (B) Docking score of Frax with different CDs. (C) 3 D binding model between Frax and CDs with different orientations as obtained from docking calculations. Top, side, and bottom views of β-CD-Frax, HP-β-CD-Frax, SBE-β-CD-Frax, and G_2_-β-CD-Frax inclusion complexes are shown. Frax is shown in green, CDs in magenta, and hydrogen bonding as a blue dashed line.

Computational molecular docking (MD) was performed to identify modeling of the interactions between CDs and Frax, thus aiding the selection of the optimal CD derivative for rational formulation design of Frax. We docked Frax (Figure 4(A)) onto the molecular surface of the CDs in different orientations. The docking score of Frax with different CDs is given in Figure 4(B). The docking scores of CD-Frax complexes followed the order β-CD > G_2_-β-CD > HP-β-CD > SBE-β-CD, suggesting the high-affinity of β-CD and G_2_-β-CD for Frax binding. Images of Frax docked into different CDs are shown in Figure 4(C) and Figure S1. The oxygen atom of the ester group in Frax forms a hydrogen bond with the hydroxyl group in all the CDs, which helps us gain deep insights into the host-guest interactions of the complexes.

### 3.4. *In vitro* release of Frax from cylcodextrin complexes

**Figure 5.**
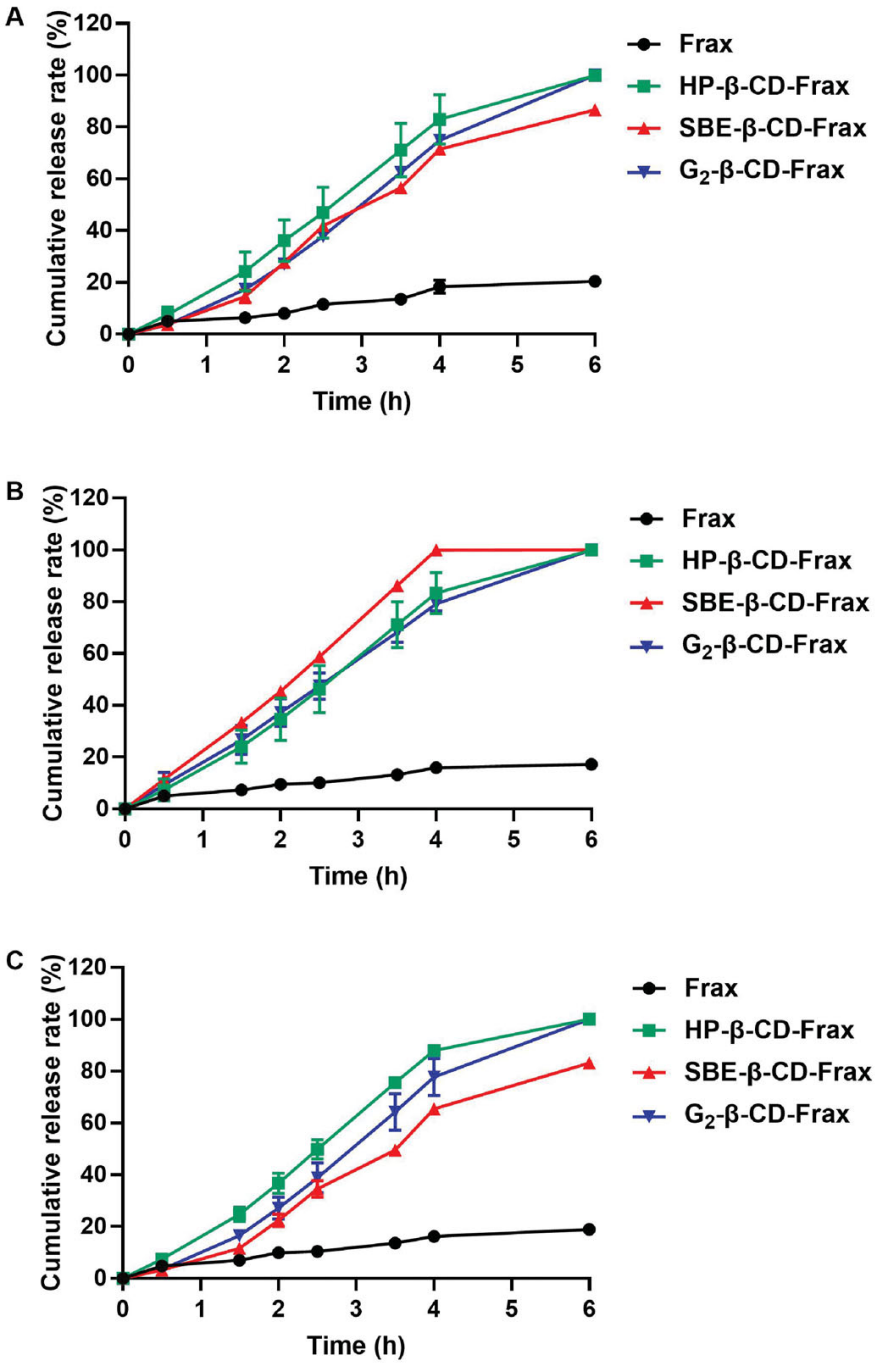
Release of fraxinellone (Frax) from CD-Frax complexes *in vitro*. Free Frax or CD-Frax complexes was dissolved at 37 °C in artificial gastric juice (A) (0.1 M HCl, pH 1.2) or phosphate-buffered saline at pH 6.8 (B) or 7.4 (C). Data are mean ± *SD* (*n* = 3 per group).

All CDs-Frax achieved much faster drug release than Frax suspension regardless of the release medium used (Figure 5). The cumulative drug release from these CDs-Frax reached 80–100% within 6 h in the different simulated GI fluids. In sharp contrast, the cumulative drug release from Frax suspension was <20% during 6 h. The rapid dissolution of Frax from CDs-Frax may be due to the excellent drug solubility and loss of crystallinity of Frax in the complex, as identified in the physicochemical characterizations.

### 3.5. Cell-based studies

**Figure 6.**
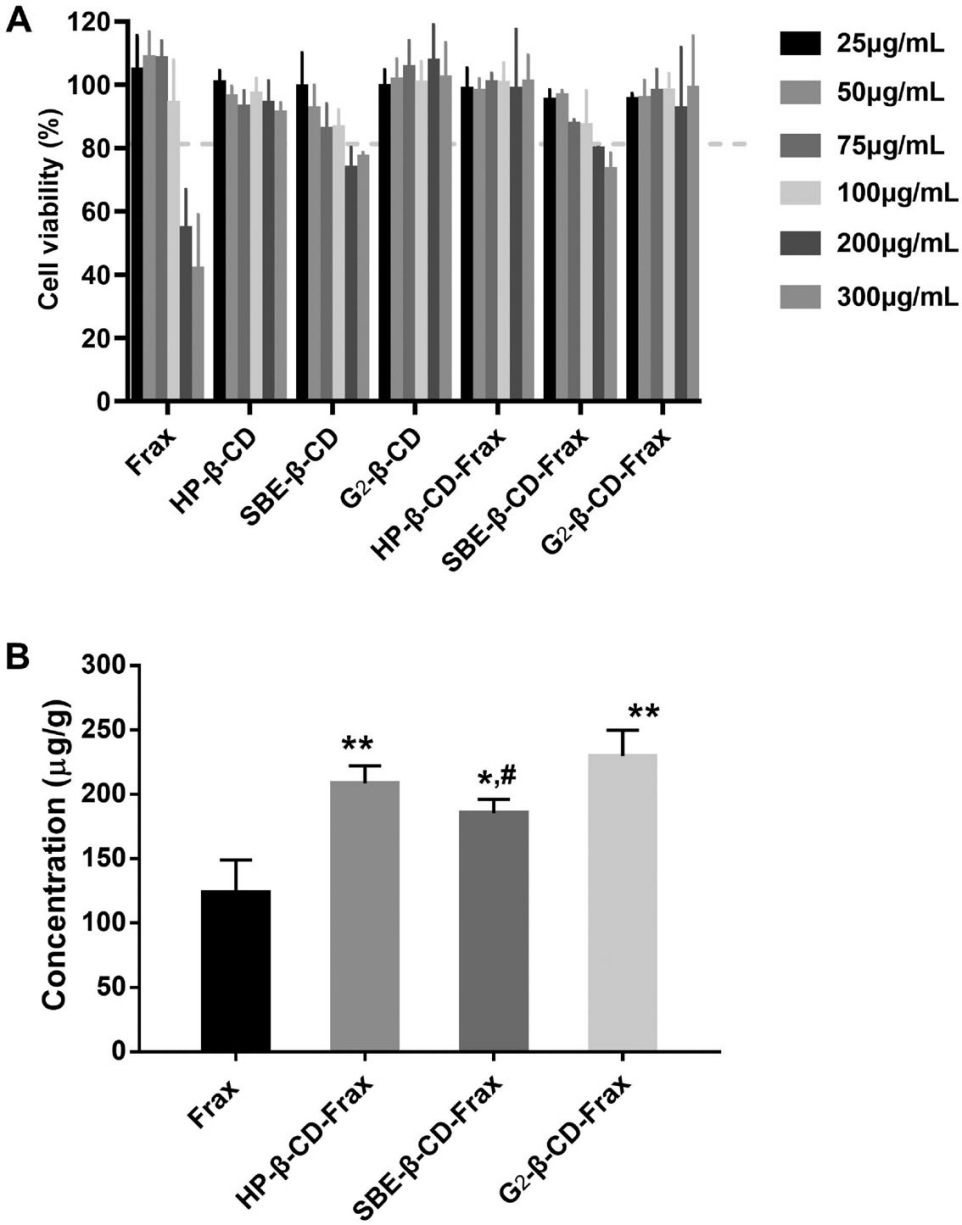
*In vitro* cell-based studies. (A) Cell viability after treatment with free Frax, CDs, or CD-Frax complexes for 4 h. (B) Cell uptake ability of free Frax, CDs, or CD-Frax complexes at a Frax concentration of 100 μg/mL 2 h post-drug treatment. **p < .01, *p < .05, compared with free Frax. *^#^p* < .05, compared with G_2_-β-CD-Frax. Data are mean ± SD (*n* = 3 per group).

We evaluated the toxicity of free Frax, CDs, or CDs-Frax against Caco-2 cells in culture. The results showed that both SBE-β-CD and SBE-β-CD-Frax at high drug concentrations (200 and 300 μg/mL) led to similar cell viability that is slightly lower than 80% (Figure 6(A)), which is probably brought about by SBE-β-CD (Mohandoss et al., 2019). It is noticeable that free Frax showed marked cell death when concentration increased to 200 μg/mL. While at the same time, over 85% of Caco-2 cells remained viable in the presence of HP-β-CD, G_2_-β-CD, and their CDs-Frax (300 μg/mL), indicating a little cytotoxic effect on cell growth. Therefore, the high concentration of Frax related cell death may be explained by the high content of cosolvents to help drug solubilize in the culture medium. Cell uptake ability studies (Figure 6(B)) showed that HP-β-CD-Frax, SBE-β-CD-Frax, G_2_-β-CD-Frax (100 μg/mL) significantly promoted the internalization of Frax into Caco-2 cells by 1.7, 1.5, and 1.8 times, respectively, indicating improved cell absorption of Frax along with increased water solubility.

### 3.6. Pharmacokinetic studies

**Figure 7.**
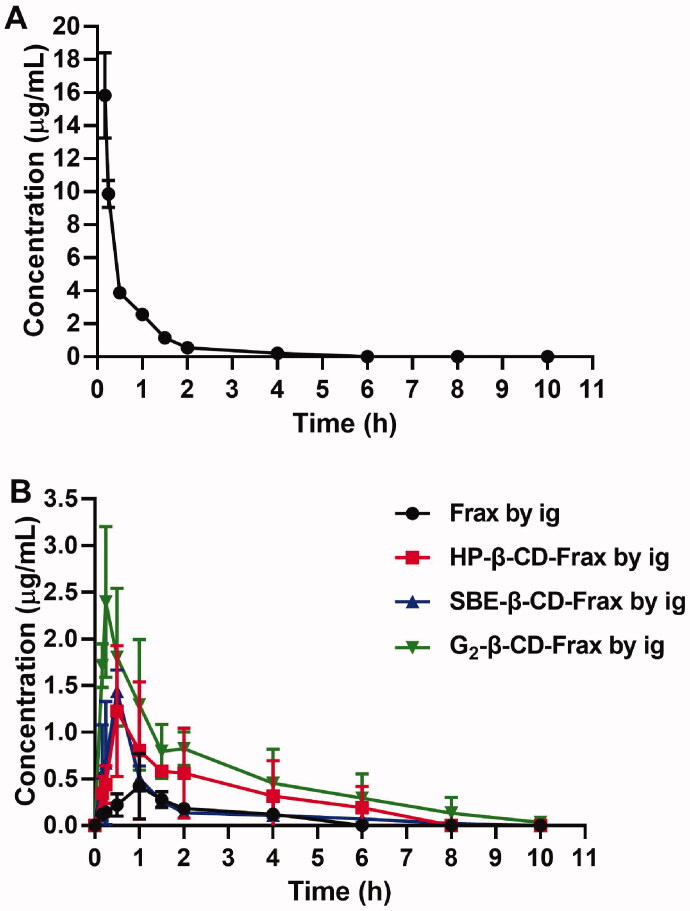
*In vivo* pharmacokinetic study. Mean plasma fraxinellone (Frax) concentration as a function of time in rats after intravenous injection of Frax (A) (10 mg/kg) or oral administration of Frax (150 mg/kg) or CDs-Frax (B) (150 mg/kg). Data shown are mean ± SD (*n* = 5 animals per group).

Before the pharmacokinetic studies, the developed HPLC method was validated for linear range, precision, and recovery. Good linearity (*R*^2^ of 0.9993) was obtained in the range of 0.05–10 μg/mL (Linearity equation: Concentration = 0.012 Peak area + 0.156). Inter-day precisions, intra-day precisions, and extraction recovery are summarized in Table S2. The validated HPLC method was applied to determine the plasma Frax concentration in the pharmacokinetic studies. To calculate the absolute bioavailability of Frax and CDs-Frax after oral administration, the plasma concentration of Frax after intravenous administration is monitored and shown in Figure 7(A). The pharmacokinetic curves and pharmacokinetic parameters of Frax and CDs-Frax inclusion complexes were shown in Figure 7(B) and Table 1, respectively. All CDs-Frax improved the oral drug absorption to some extent. Although all the CDs significantly increased the solubility of Frax, they demonstrated different oral absorption profiles and extent of oral absorption improvement. Specifically, SBE-β-CD-Frax and G_2_-β-CD-Frax showed significantly lower *T*_max_ and higher *C*_max_ than free Frax, respectively. The improved and fast absorption behavior of these two groups were mainly attributed to the higher solubility and faster release of Frax from CDs-Frax than free Frax as evidenced by solubility and *in vitro* release studies. In addition, Fr for HP-β-CD-Frax, SBE-β-CD-Frax, G_2_-β-CD-Frax was about 390, 180, and 580%, respectively. The results indicated that G_2_-β-CD-Frax had better oral drug absorption compared to other CDs-Frax. In addition, G_2_-β-CD-Frax had significantly higher cellular uptake than SBE-β-CD-Frax (^#^*p* < .05), as demonstrated by cellular uptake studies. Previous studies have suggested that the absorption enhancement of cyclodextrin complex was not impacted solely by their solubilization capacity but also by membrane permeability (Miller & Dahan, 2012; Aihara et al., 2021). Taken together, we speculated that the highest oral absorption of G_2_-β-CD-Frax was attributed not only to the improved drug solubility but also to the increase of Frax permeability through the intestinal absorption barrier. The excellent increased oral absorption of Frax by G_2_-β-CD-Frax combined with the good biocompatibility of G_2_-β-CD (Li et al., 2015), lead us to propose this CD derivative as a drug delivery system and investigate its therapeutic efficacy in following pharmacodynamic studies.

**Table 1.** Main pharmacokinetic parameters of free Frax in rats after intravenous injection (10 mg/kg) and after oral administration of Frax or CDs-Frax (150 mg/kg) (*n* = 5 animals per group).

Parameters	Frax (i.v.)	Frax (p.o.)	HP-β-CD-Frax (p.o.)	SBE-β-CD-Frax (p.o.)	G_2_-β-CD-Frax (p.o.)
AUC_0–∞_ (μg/mL*h)	11.81 ± 1.43	0.97 ± 0.23	3.44 ± 1.82*	1.69 ± 0.33**	5.33 ± 1.71***
*C*_max_ (μg/mL)	–	0.49 ± 0.29	1.45 ± 0.56*	1.55 ± 0.08***	2.48 ± 0.66***
*T*_max_ (h)	–	1.00 ± 0.35	1.50 ± 1.70	0.44 ± 0.13*	0.30 ± 0.11**
*F_a_* (%)	–	0.50	1.90	0.95	2.90
*F_r_* (%)	–	–	390.00	180.00	580.00

AUC: area under the plasma concentration-time curve; *C*_max_: maximum plasma concentration; *T*_max_: time taken to reach *C*_max_.

*p < .05, **p < .01, ***p < .001 compared with Frax (p.o.) group.

### 3.7. *In vivo* therapeutic efficacy of G2-β-CD-Frax against hepatic fibrosis

**Figure 8.**
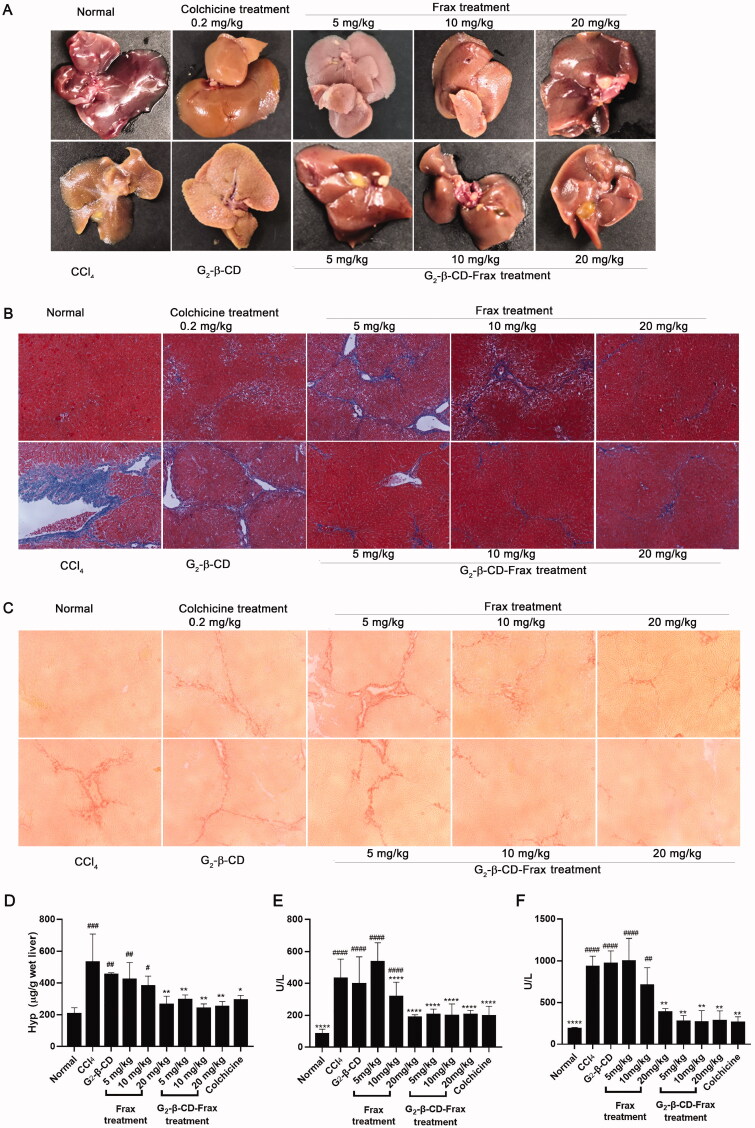
G_2_-β-CD-Frax improved anti-fibrosis efficacy of Frax on the CCl_4_-induced mouse model. Representative photos of liver tissues (A) and images of liver sections with Masson (B) and Sirius red staining (C). (Scale bars, 100 μm) (D) Concentration of liver HYP. (E) ALT and (F) AST levels in serum. ****p < .0001, **p < .01, *p < .05, compared with the CCl_4_-induced group. *^####^p* < .0001, *^###^p* < .001,*^##^p* < .01, *^#^p* < .05, compared with normal group. Data are mean ± *SD* (*n* = 3 per group).

Finally, we investigated the therapeutic effect of orally administered free Frax and G_2_-β-CD-Frax on CCl_4_-induced liver fibrosis mice. Subcutaneously injected CCl_4_ in olive oil was used to establish a hepatic fibrosis model in mice followed by scheduled drug administration (Yan et al., 2019). To better demonstrate the therapeutic effect of G_2_-β-CD-Frax on liver fibrosis, CCl_4_-induced liver fibrosis mice were orally given free Frax and G_2_-β-CD-Frax at three different doses of 5, 10, and 20 mg/kg, respectively. At the end of the experiment, we assessed liver tissue morphology by visual inspection, levels of major biochemical indicators, Masson and Sirius Red staining of paraffin-embedded liver sections (Figure 8). CCl_4_ treatment obviously impaired the liver tissue structure and resulted in collagen deposition and rough liver surface, as indicated by Figures 8(A–C). Compared with the CCl_4_ treatment group, G_2_-β-CD-Frax treatment at a low dose of 5 mg/kg remarkably improved liver appearance, alleviated collagen accumulation, and reduced levels of crucial liver fibrosis markers involving AST, ALT, and HYP (Figure 8, *p* < .05), which were as low as the normal group (*p* > .05). Meanwhile, a similar decrease of these mediators and slight collagen deposition were detected in the G_2_-β-CD-Frax treated mice (10 and 20 mg/kg). In sharp contrast, the free Frax group (5 mg/kg) exhibited no significant decrease in the level of these biomarkers and no obvious inhibition on the collagen fibers production in liver tissue compared with the CCl_4_ group, as shown in histological staining and hepatic tissue morphology pictures. Even when the Frax dose increased to 20 mg/kg, free Frax alone only showed a rather modest effect on liver fibrosis. Collectively, these results suggested that G_2_-β-CD-Frax showed superior efficacy to free Frax for liver fibrosis.

### 3.8. Safety evaluation of orally administered G_2_-β-CD and G_2_-β-CD-Frax

In addition to therapeutic efficiency, toxicity is another crucial factor in the application of therapeutic agents. The systemic toxicity of *G_2_-β-CD and G_2_-β-CD-Frax* after oral administration was detected. H&E staining results demonstrated no inflammation and no cellular damage induced by *G_2_-β-CD and G_2_-β-CD-Frax* (Figure S2). In addition, no obvious changes were observed in the blood biochemical assay (Table S3). Taken together, these results suggested the good biosafety of *G_2_-β-CD* as a promising delivery vehicle for oral administration.

## 4. Conclusion

The formulation design of Frax through its molecular encapsulation into CDs for improved oral hepatic fibrosis therapy has been achieved. G_2_-β-CD, one of the more recently developed hydrophilic CD derivatives, can complex more stably with Frax and exhibited superior solubilizing capacity than other CDs tested. Moreover, G_2_-β-CD-Frax exhibited excellent drug absorption improvement both *in vitro* and *in vivo*. Orally administered G_2_-β-CD-Frax (5 mg/kg) enabled efficient treatment for liver fibrosis without inducing toxicity. Our study is the first report to validate the utilization of G_2_-β-CD in oral delivery of drugs poorly soluble in water, like Frax, for improved therapeutic effect.

## Supplementary Material

Supplemental Material
