# Supplementary material for: Rational formulation engineering of fraxinellone utilizing 6-O-α-D-maltosyl-β-cyclodextrin for enhanced oral bioavailability and hepatic fibrosis therapy
Source: Drug Deliv. 2021 Sep 14;28(1):1890–902. doi: 10.1080/10717544.2021.1976310 (PMC8451604; doi:10.1080/10717544.2021.1976310)
Supplement: Supplemental Material [file IDRD_A_1976310_SM8977.docx]

**Supplementary material**

Rational formulation engineering of Fraxinellone utilizing 6-O-α-D-maltosyl-β-cyclodextrin for enhanced oral bioavailability and hepatic fibrosis therapy

Jianbo Li ^a^, Tiange Feng ^b,c^, Weijing Yang ^b,c^, Yaru Xu ^b,c^, Shuaishuai Wang ^b,c^, Huijie Cai ^b,c^, Zhilei Liu ^a^, Hong Qiang ^b,c^ , Jinjie Zhang ^b,c,^*

^a^ Institute of Medical and Pharmaceutical Sciences, Zhengzhou University, Zhengzhou, Henan Province 450052, China.

^b^ Henan Key Laboratory of Targeting Therapy and Diagnosis for Critical Diseases, School of Pharmaceutical Sciences, Zhengzhou University, Zhengzhou, Henan Province 450001, China

^c^ Collaborative Innovation Center of New Drug Research and Safety Evaluation, Zhengzhou 450001, Henan Province, China.

* Corresponding author: Jinjie Zhang*

Mailing address: No. 100 Kexue Road, Zhengzhou, Henan Province 450001, China. Tel: +86-371-67781908.

Email address: [liger1029@126.com](mailto:liger1029@126.com)

**Methods**

**HPLC**

Frax concentration was determined using a Waters 2690 HPLC system coupled to a Photodiode Array Detector (USA). An analytical column (Zorbax C_18_ column of 150 mm × 4.6 mm) was used. Water and methanol (35:65, v/v) were used as the mobile phase and the flow rate was 1.0 mL/min. Frax was detected at 220 nm with a sampling volume of 10 μL. The assay was linear over the range of 0.5-60 μg/mL based on the regression equation: concentration = 0.00005 Peak area + 0.2945 (r = 0.9999).

**FTIR**

The FTIR spectra of the individual compound and CD-Frax inclusion complexes were recorded using a Thermo Fisher Scientific FTIR spectrophotometer (Nicolet is 10, USA). The FTIR measurements were performed in the scanning range of 400 to 4000 cm^-1^ at room temperature.

**P-XRD**

Powder X-ray diffraction patterns of the individual compound and CD-Frax inclusion complexes were obtained using a powder X-ray diffractometer (Bruker, D8 ADVANCE, Germany), with a copper target X-ray tube set to 40 kV and 40 mA with Cu Kα radiation. All samples were measured in the 2θ range of 5 °C-80 °C at a scan rate of 0.1 sec/step.

**Thermal analysis**

The thermal behavior of individual compound and CD-Frax inclusion complexes has been studied by simultaneous DSC/TGA (TA Instruments, SDT Q600 V20.9 Build 20, USA). The sample (~5 mg) was accurately weighed into an aluminum pan. Samples were heated from 20 °C to 400 °C at a rate 10 °C/min under a nitrogen atmosphere.

**FESEM**

The morphology of the individual compound and CD-Frax inclusion complexes were investigated by FESEM using a high-resolution microscope (FEI, Quanta 400 FEG, USA) at 20 kV in high-vacuum mode. Sample preparation was minimal, it consisted of immobilizing on a conductive adhesive with further gold plating.

**Results:**

Table S1. ^1^H NMR chemical shifts (δ, ppm) in different formulations of fraxinellone (Frax) and the difference from the corresponding shift in free Frax (δ), as determined in dimethyl sulfoxide (DMSO)-d_6_.

| **Formulation** |  |  | **Proton** |  |  |
| --- | --- | --- | --- | --- | --- |
|  | **H-1** | **H-2** | **H-3** | **H-4** | **H-5** |
| Frax | 0.7784 | 5.0027 | 7.6973 | 7.7184 | 6.5051 |
| β-CD-Frax (δ) | 0.7755 | 5.0023 | 7.6967 | 7.7158 | 6.5047 |
| Δδ | -0.0029 | -0.0004 | -0.0006 | -0.0026 | -0.0004 |
| HP-β-CD-Frax (δ) | 0.7753 | 4.7709 | 7.6933 | 7.7129 | 6.5012 |
| Δδ | -0.0031 | -0.2318 | -0.0040 | -0.0055 | -0.0039 |
| SBE-β-CD-Frax (δ) | 0.7753 | 5.0021 | 7.6956 | 7.7170 | 6.5008 |
| Δδ | -0.0031 | -0.0006 | -0.0017 | -0.0014 | -0.0043 |
| G_2_-β-CD-Frax (δ) | 0.7748 | 5.0002 | 7.6967 | 7.7167 | 6.5051 |
| Δδ | -0.0036 | -0.0025 | -0.0016 | -0.0017 | 0 |

Table S2. Precision and extraction recovery for Frax in rat plasma.

| Sample concentration (μg/mL) | Inter-day precision | Intra-day precision | Extration recovery (%) |
| --- | --- | --- | --- |
| 0.20 | 6.08% | 7.28% | 86.63% ± 4.06 |
| 3.33 | 4.55% | 8.95% | 90.10% ± 5.91 |
| 8.00 | 3.92% | 4.41% | 92.05% ± 3.16 |

Table S3. Results of Blood biochemical assay. (n=10)

| Hematological parameters | Blank | Model group | G_2_-β-CD-Frax | Frax  (5 mg/kg) | Frax  (10 mg/kg) | Frax  (20 mg/kg) | G_2_-β-CD-Frax  (5 mg/kg) | G_2_-β-CD-Frax  (10 mg/kg) | G_2_-β-CD-Frax  (20 mg/kg) | Colchicine |
| --- | --- | --- | --- | --- | --- | --- | --- | --- | --- | --- |
| WBC | 4.47 ± 0.78 | 8.55 ± 1.95 | 8.17 ± 4.34 | 7.78 ± 3.91 | 6.46 ± 2.46 | 8.32 ± 3.90 | 7.06 ± 2.58 | 7.53 ± 3.25 | 9.27 ± 3.88 | 9.61 ± 4.41 |
| RBC | 9.50 ± 0.41 | 7.85 ± 2.31 | 8.95 ± 0.88 | 9.21 ± 0.75 | 8.42 ± 0.32 | 9.07 ± 0.33 | 8.45 ± 0.45 | 8.72 ± 0.87 | 8.59 ± 0.61 | 9.13 ± 1.01 |
| HGB | 138.33 ± 2.31 | 130.17 ± 11.30 | 130.75 ± 12.28 | 135.50 ± 10.76 | 127.00 ± 5.10 | 131.50 ± 5.66 | 128.88 ± 6.36 | 130.75 ± 14.74 | 125.50 ± 9.99 | 133.57 ± 9.50 |
| HCT | 41.40 ± 0.82 | 34.73 ± 9.74 | 38.26 ± 3.99 | 39.54 ± 2.28 | 37.15 ± 1.70 | 38.83 ± 1.69 | 37.85 ± 2.05 | 38.09 ± 4.30 | 36.50 ± 2.59 | 39.06 ± 2.62 |
| MCV | 43.58 ± 2.09 | 43.63 ± 2.06 | 43.51 ± 2.53 | 43.01 ± 2.17 | 43.80 ± 1.44 | 42.83 ± 1.54 | 44.81 ± 1.98 | 43.66 ± 1.86 | 42.50 ± 1.66 | 43.09 ± 2.97 |
| MCH | 14.75 ± 0.80 | 14.81 ± 0.42 | 14.65 ± 0.61 | 14.73 ± 0.49 | 15.09 ± 0.46 | 14.51 ± 0.46 | 15.28 ± 0.74 | 14.98 ± 0.63 | 14.61 ± 0.49 | 14.71 ± 0.70 |
| MCHC | 338.17 ± 5.98 | 340.14 ± 7.71 | 342.25 ± 12.71 | 342.63 ± 10.38 | 344.43 ± 9.64 | 338.75 ± 3.58 | 340.88 ± 7.68 | 343.25 ± 6.48 | 343.3 ± 9.09 | 341.86 ± 9.91 |
| RDW-CV | 14.48 ± 0.83 | 16.30 ± 0.86 | 15.89 ± 1.10 | 16.24 ± 1.32 | 16.22 ± 0.59 | 16.54 ± 0.79 | 16.83 ± 1.24 | 15.89 ± 0.90 | 16.01 ± 1.14 | 16.24 ± 1.60 |
| RDW-SD | 26.60 ± 1.82 | 27.67 ± 1.15 | 28.88 ± 2.03 | 29.88 ± 3.52 | 30.71 ± 1.70 | 30.13 ± 2.23 | 31.71 ± 2.29 | 29.38 ± 2.26 | 29.00 ± 2.93 | 29.86 ± 4.02 |
| PLT | 965.83 ± 361.88 | 1020.43 ± 376.88 | 1415.57 ± 240.16 | 1188.13 ± 317.24 | 1012.71 ± 281.88 | 1190.00 ± 157.80 | 1312.75 ± 234.34 | 1114.38 ± 322.86 | 1191.88 ± 269.40 | 1363.86 ± 333.63 |
| MPV | 6.40 ± 0.58 | 6.66 ± 0.52 | 6.54 ± 0.23 | 6.51 ± 0.32 | 6.63 ± 0.44 | 6.38 ± 0.23 | 6.46 ± 0.44 | 6.83 ± 0.48 | 6.70 ± 0.69 | 6.80 ± 0.51 |
| PDW | 15.03 ± 0.31 | 15.04 ± 0.26 | 14.90 ± 0.10 | 14.84 ± 0.12 | 14.91 ± 0.12 | 14.86 ± 0.17 | 14.98 ± 0.27 | 15.00 ± 0.18 | 14.94 ± 0.20 | 14.99 ± 0.22 |

Abbreviation: WBC, white blood cell; RBC, red blood cell; Plt, platelet; HGB, hemoglobin; HCT, haematocrit; MCV, mean cell volume; MCH, mean corpuscular hemoglobin; MCHC, mean corpuscular hemoglobin concentration; RDW-CV, red cell volume distribution width; PLT, platelet; PCT, procalcitonin; MPV, meanplateletvolume; PDW, platelet distribution width.

**
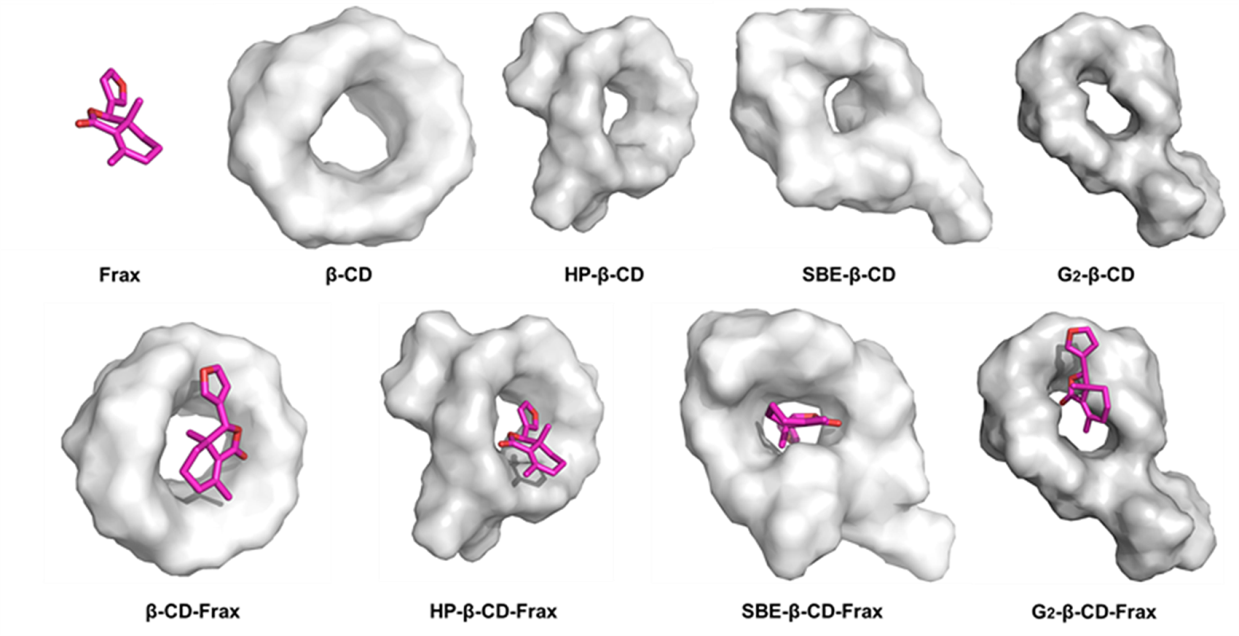
**

Figure S1. Moledular dynamics simulation models for Frax, CDs and Frax-CDs, respectively.

**
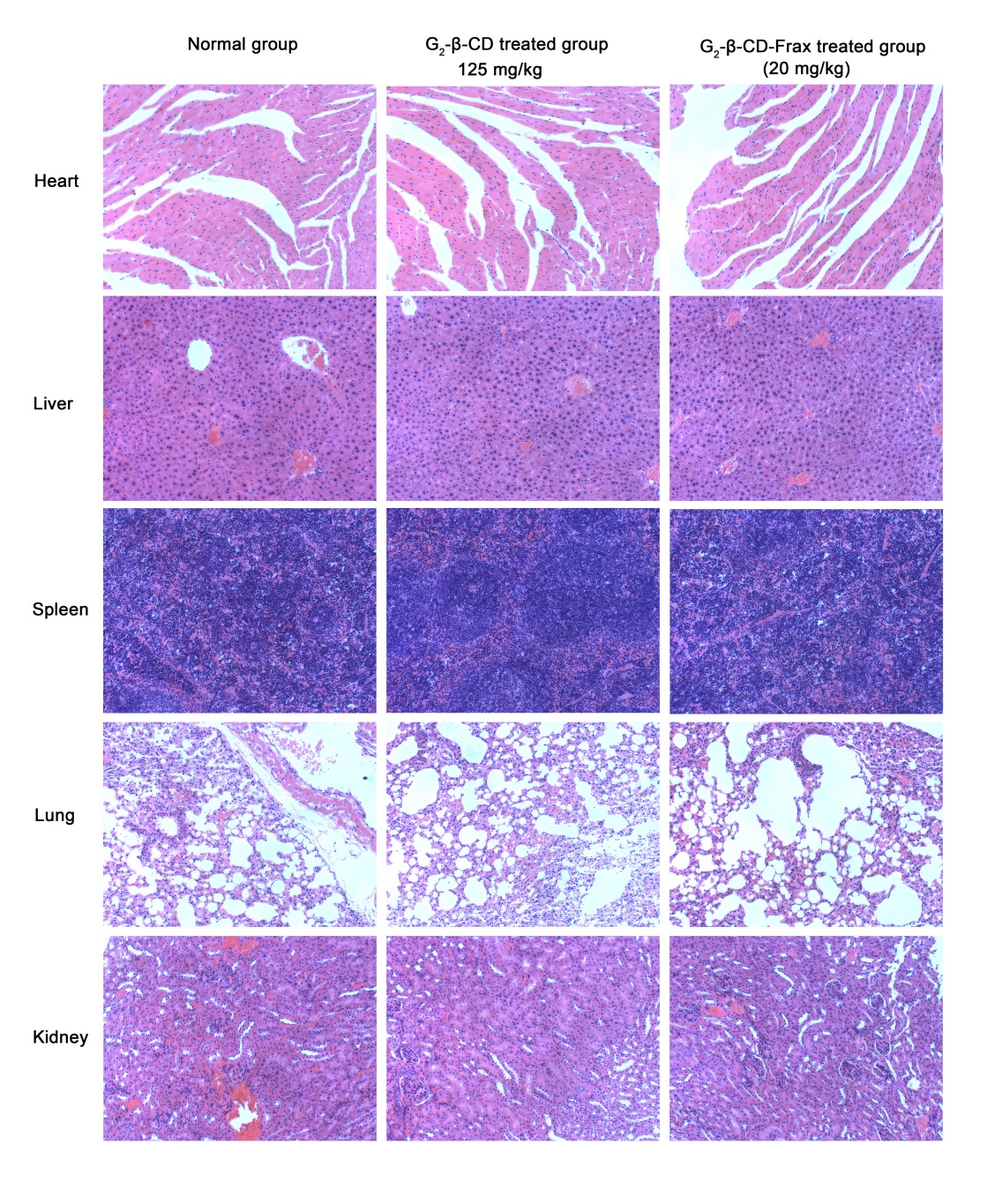
**

Figure S2. Histopathologic examination of mice’s major tissues after long-term treatment of G_2_-β-CD and G_2_-β-CD-Frax.
